# Supplementary material for: Apolipoprotein A-IV binds αIIbβ3 integrin and inhibits thrombosis
Source: Nat Commun. 2018 Sep 6;9:3608. doi: 10.1038/s41467-018-05806-0 (PMC6127106; doi:10.1038/s41467-018-05806-0)
Supplement: Supplementary file 1 — Supplementary Information [file 41467_2018_5806_MOESM1_ESM.pdf]

Supplementary Figure 1

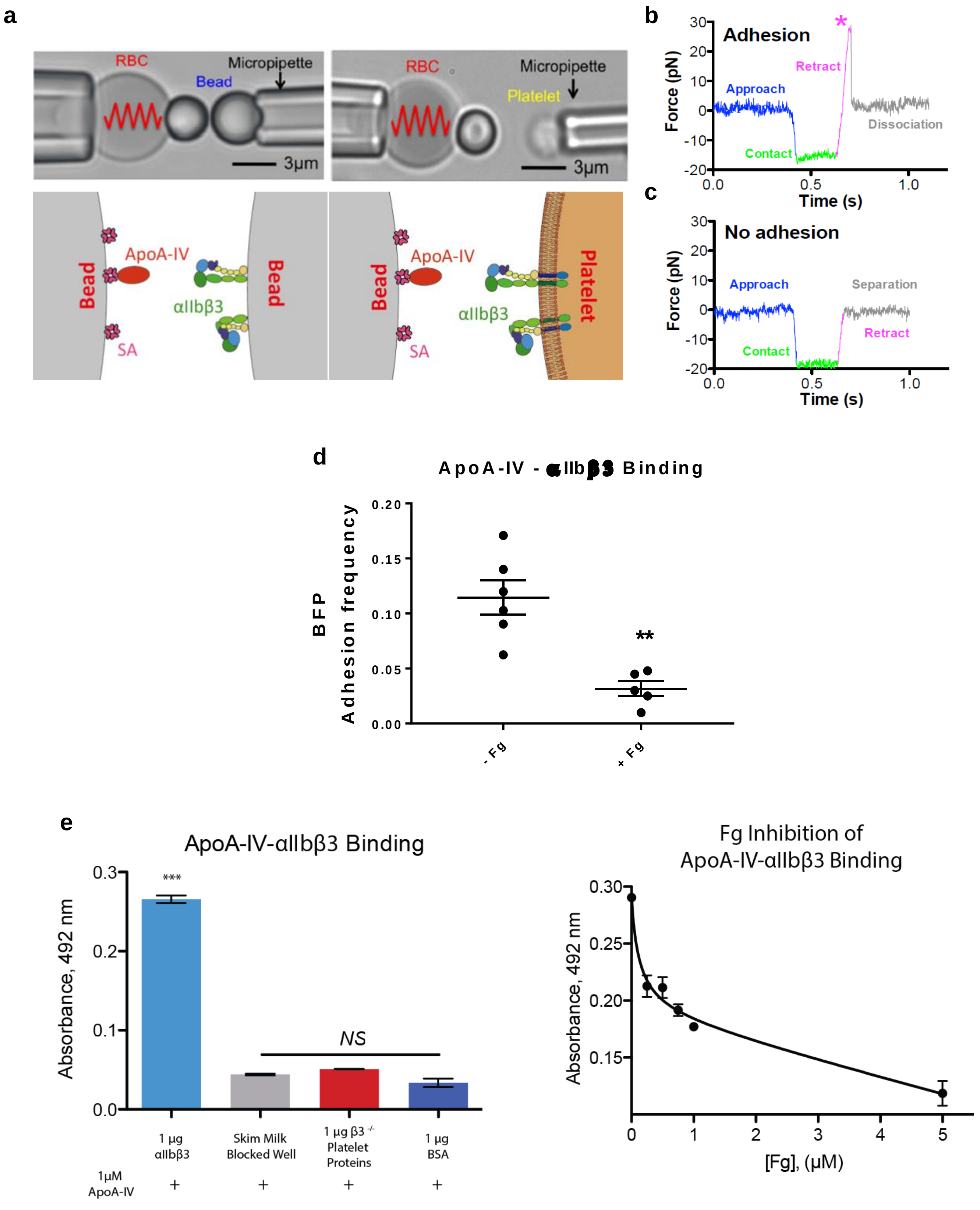

a, Biomembrane-Force-Probe (BFP) detection of apoA-IV-αIIbβ3 integrin binding in a purified (***left***) and a native platelet (***right***) systems (RBC, red blood cell; SA, streptavidin). **b**, **c**, Representative BFP traces for adhesion (**b;** * indicates apoA-IV-αIIbβ3 adhesion) and no adhesion events (**c**). **d-e**, Fibrinogen (Fg) inhibited apoA-IV binding to αIIbβ3 as determined by BFP adhesion frequency assay (**d**) and ELISA (**e**). **d**, The apoA-IV-αIIbβ3 adhesion frequencies in a purified system were measured in the absence and presence of Fg (0.3µM) in solution. n ≥ 5 probe–target pairs per group. **e**, The inhibition of varying concentrations of Fg (0-5 µM) on apoA-IV (1 µM) binding to immobilized αIIbβ3 (1 µg). Control experiments (***left***) confirmed that the ELISA signal observed was from specific binding interactions. The concentration-response curves were fit to the half maximal inhibitory model (methods) to determine the IC_{50} for both Fg and apoA-IV. The IC_{50} for Fg inhibition of apoA-IV- αIIbβ3 was determined to be 0.123 ± 0.061 µM. The inhibition data is consistent with the BFP data as it shows that the affinity of apoA-IV-αIIbβ3 is less than that of Fg-αIIbβ3. It is worth noting that difference between the IC_{50} values cannot be compared directly to the difference of the BFP affinity values as the IC_{50} values are non-linearly dependent on the concentration of ligand inhibited. n=4. Assessed by unpaired, two-tailed Student's t-test. ** P< 0.01 vs. control. Mean ± SEM.

Supplementary Figure 2

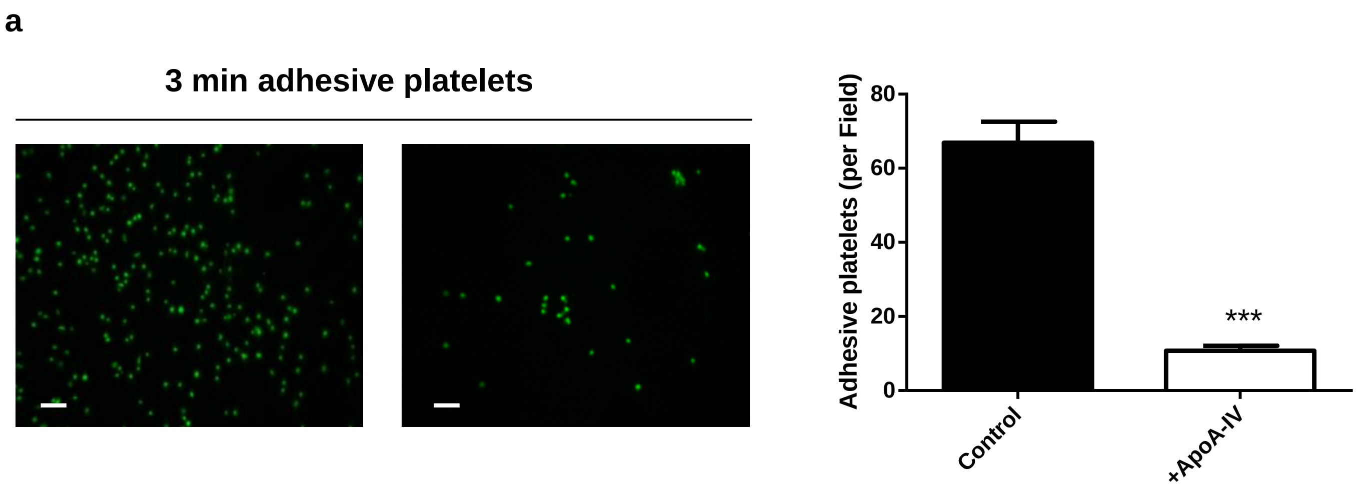

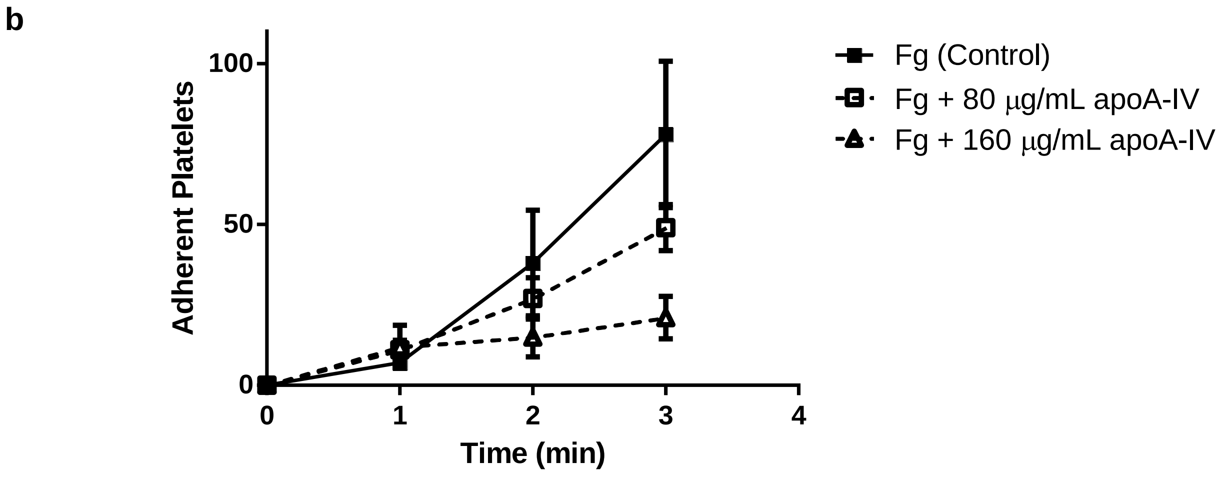

a, Representative images of adhesive platelets (3 min perfusion). **b**, Adherent platelets were enumerated at 0, 1, 2, and 3 min of perfusion at 600 s^{-1}. Increasing doses of apoA-IV resulted in an enhanced ability to block platelet deposition on an immobilized Fg matrix. BSA was used as control. n=4. Assessed by unpaired, two-tailed Student's t-test. *** $P < 0.001$. Mean ± SEM. Scale bars: 10 μm (**a**)

Supplementary Figure 3

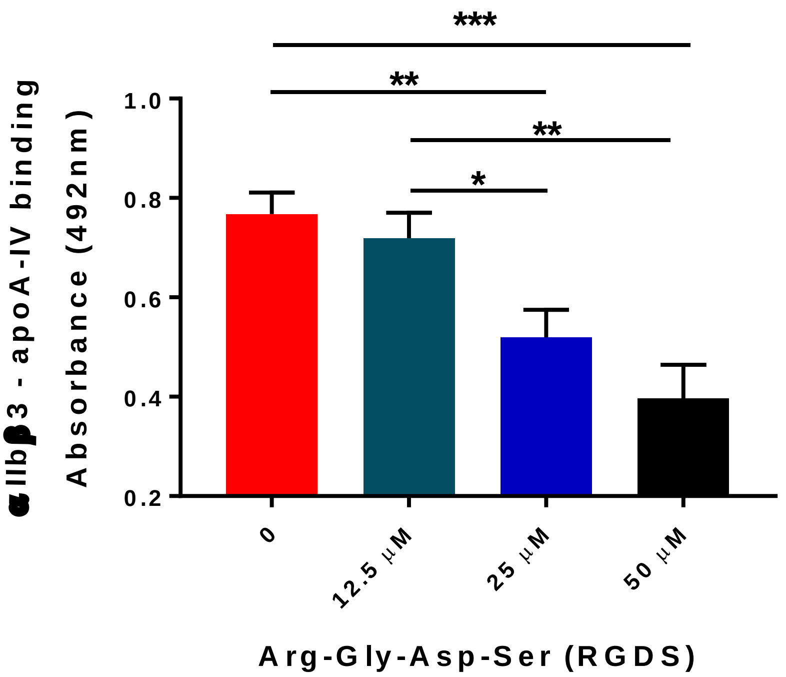

Arg-Gly-Asp-Ser (RGDS) inhibited apoA-IV binding to αIIbβ3 in ELISA. The inhibition of varying concentrations of RGDS (0-50µM) on apoA-IV (160µg/mL) binding to immobilized αIIbβ3 (1 µg) was shown. n=7. Assessed by unpaired, two-tailed Student's t-test. * $P < 0.05$; ** $P < 0.01$; *** $P < 0.001$. Mean ± SEM.

Supplementary Figure 4

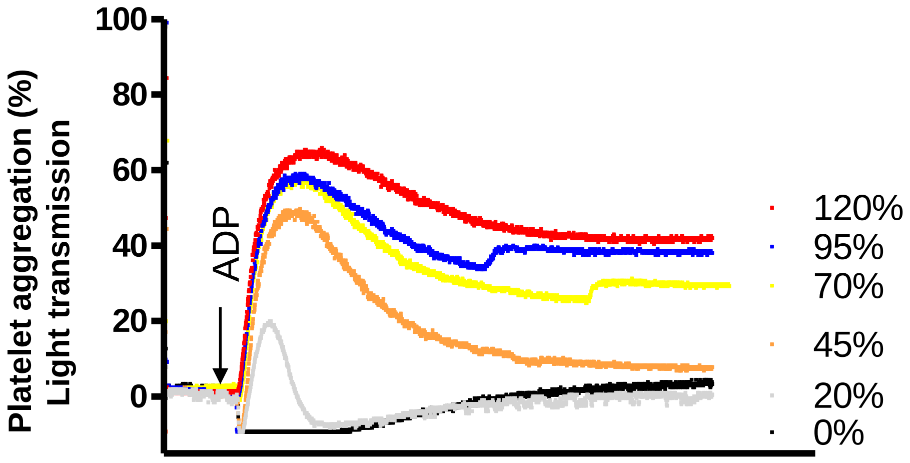

Anti-coagulated blood was collected from $Fg^{-/-}$ mice and different concentrations of Fg (100 % = 1.5 mg/mL) were added into $Fg^{-/-}$ PRP. Platelet aggregation was induced by ADP. n=3.

Supplementary Figure 5

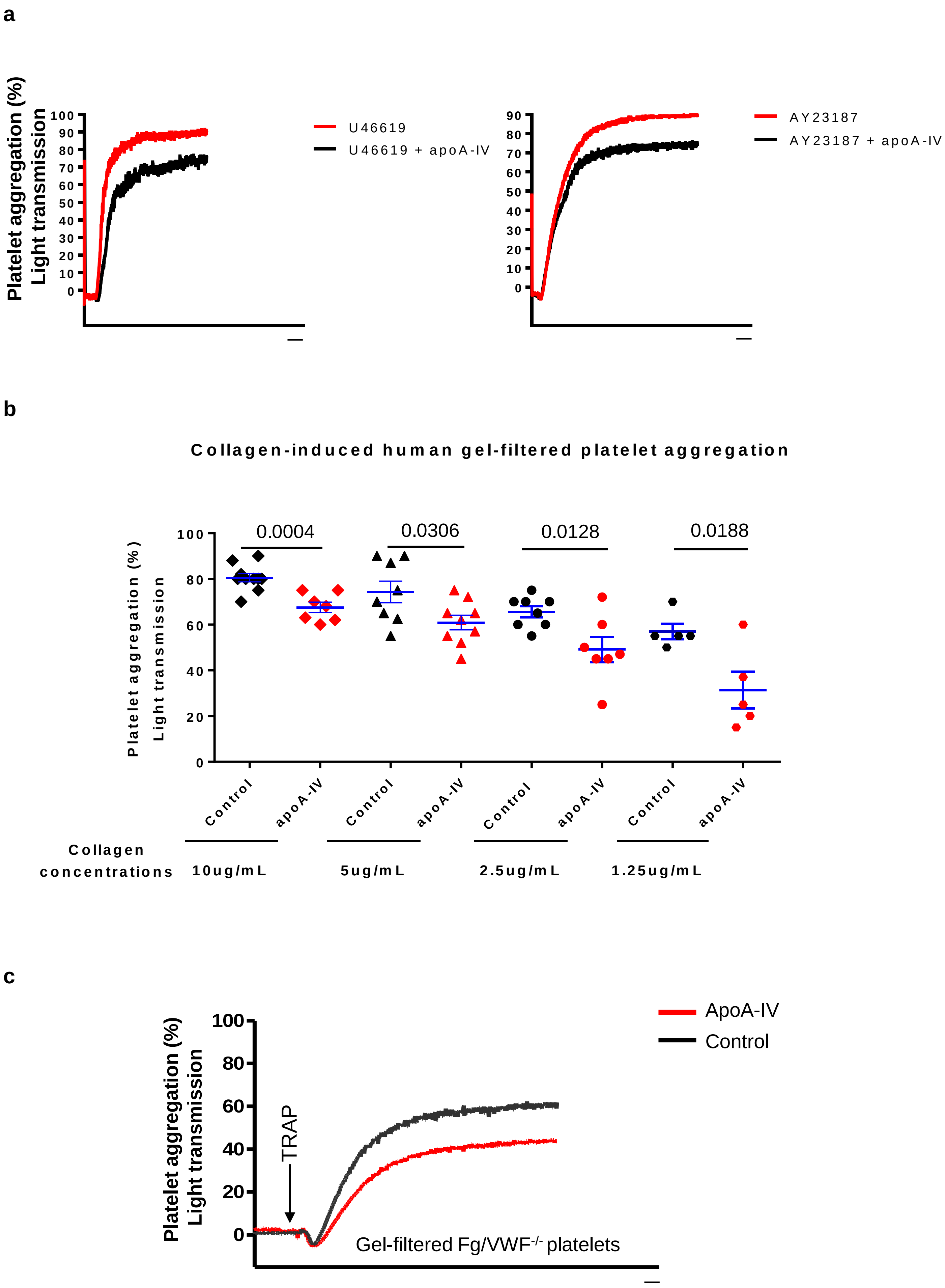

a, Recombinant apoA-IV reduced thromboxane A_2 analogue (U46619, 25 µM)- and calcium ionophore (AY23187, 25 µM)-induced human platelet aggregation in platelet-rich plasma. PBS was used as control (n=4). **b**, Recombinant human apoA-IV (160µg/mL) inhibited human platelet aggregation induced by different doses of collagen (1.25 – 10µg/mL; P<0.05). n= 6-9. P values between treatments are indicated above groups. **c**, ApoA-IV inhibited Fg/VWF-independent platelet aggregation. Platelet aggregation in gel-filtered platelets from Fg/VWF-deficient mice were stimulated by TRAP (500 µM). Assessed by unpaired, two-tailed Student's t-test. Scale bars: 2 minute (**a** and **c**).

Supplementary Figure 6

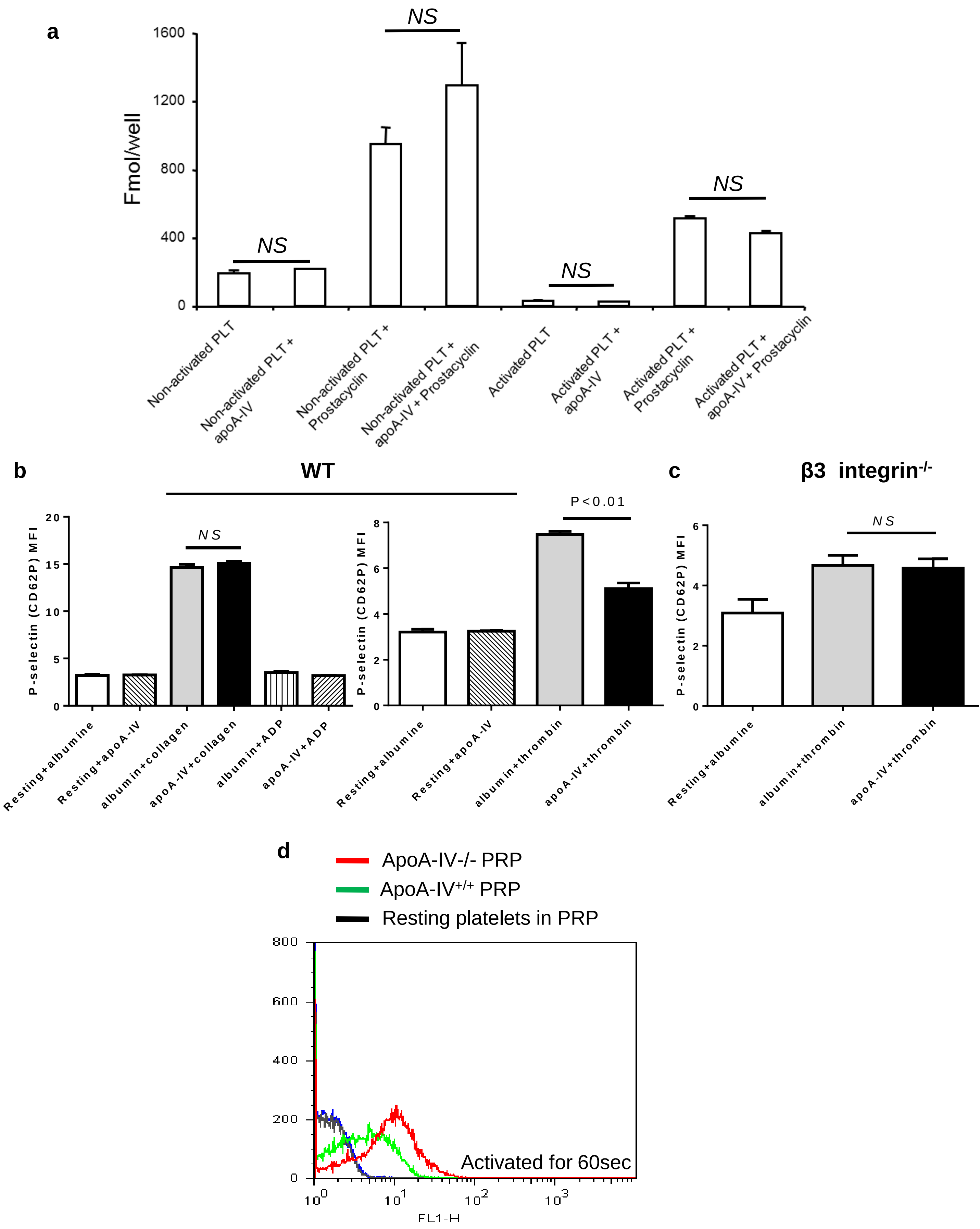

a, ApoA-IV did not significantly alter platelet cAMP levels. Platelet cAMP levels in either activated or non-activated platelets (PLT) were determined using a cAMP enzyme immunoassay (cAMP Biotrak EIA, GE Healthcare Bio-Sciences Corp, USA). Either activated or non-activated platelets (1×10^7/mL) were incubated at 37°C for 2 min and then either apoA-IV (160 µg/mL) or prostacyclin (0.3 mM) was added and allowed to incubate for an additional 2 min. After incubation, samples were centrifuged (1050 ×g, 10 min) and pellets were resuspended in 200 mL of lysis reagent. cAMP was then measured following the manufacturer's instructions. **b, c, ApoA-IV inhibited P-selectin expression on platelets depending on β3 integrin following activation.** ApoA-IV inhibited thrombin-induced P-selectin expression on WT mouse platelets (**b**) but not on β3 integrin deficient (β3 integrin$^{-/-}$) mouse platelets following thrombin activation (**c**). 4 X 10^5 WT mouse platelets and 1 X 10^6/mL gel-filtered β3 integrin$^{-/-}$ mouse platelets were incubated with recombinant mouse apoA-IV (160 µg/mL) or albumin (same molar ratio) for 10 min. The platelets were then treated with thrombin (2 U/mL), collagen (2.5 µg/mL), and ADP (40 µM) for 2 min. **d**, Significantly more P-selectin was found on the surface of platelets from apoA-IV$^{-/-}$ PRP. ApoA-IV$^{-/-}$ or apoA-IV$^{+/+}$ platelets in PRP were activated by TRAP (250 µM). P-selectin expression was detected by flow cytometry. n=3. Assessed by unpaired, two-tailed Student's t-test. NS not significant. Mean ± SEM.

Supplementary Figure 7

a

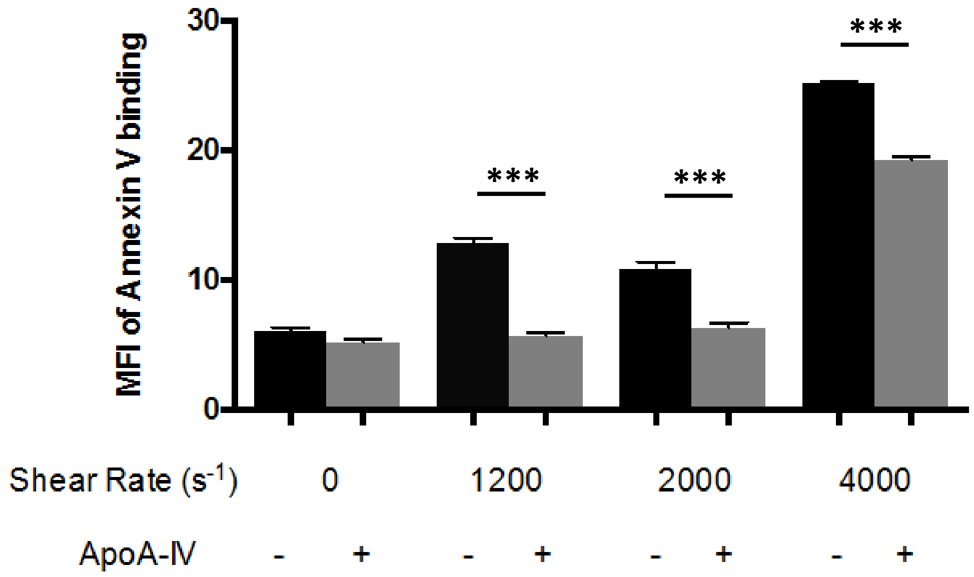

b

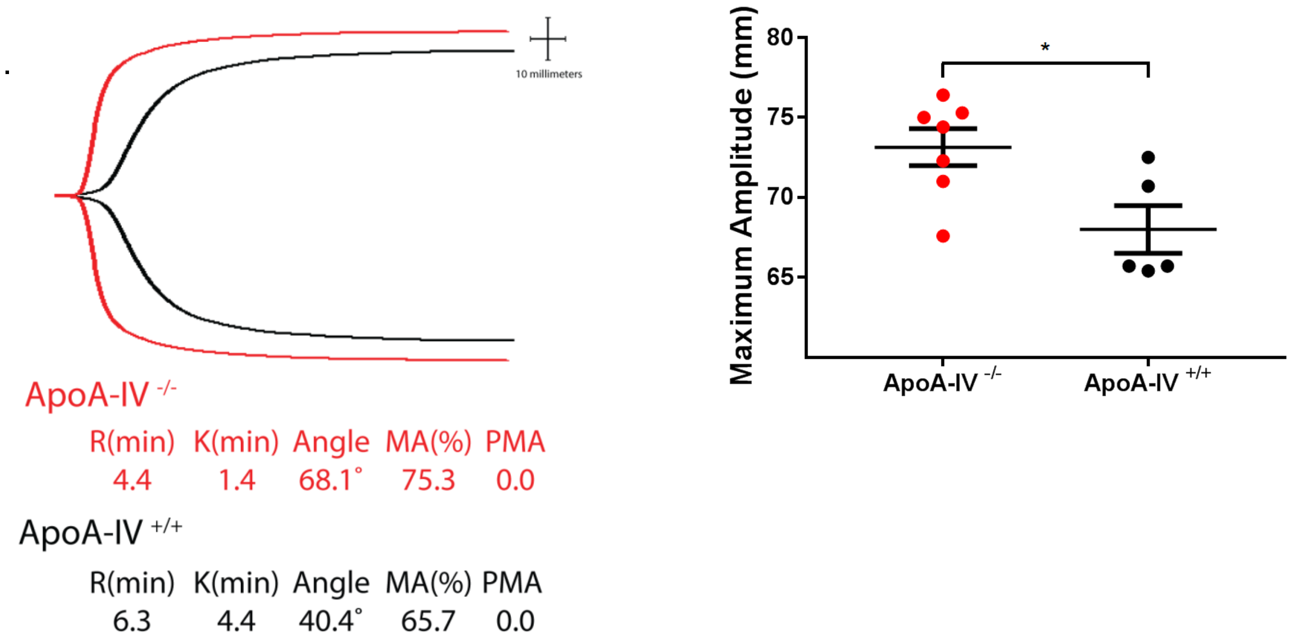

ApoA-IV inhibited shear-induced platelet PS exposure and blood coagulation. a, ApoA-IV inhibited shear-induced PS exposure. Human PRP (67μL) was subjected to different shear rates (0-4000 s^{-1}) with a cone-and-plate viscometer for 2min at 37℃. Tested samples (5μL) were then incubated with Annexin V for 30min. Annexin V binding to human platelets was detected by flow cytometry. ApoA-IV inhibited PS exposure at shear rates from 1200 s^{-1} to 4000 s^{-1}. n=3. **b**, **ApoA-IV knockout (ApoA-IV$^{-/-}$) mice have enhanced clot strength.** Representative tracing (***left***) and maximum amplitudes (***right***) of whole blood clots from apoA-IV wild-type (apoA-IV$^{+/+}$) or apoA-IV$^{-/-}$ mice as measured by thromboelastography. Blood was collected from the inferior vena cava of anesthetized mice with a 25-guage needle and immediately mixed with 3.2% sodium citrate at a 9:1 ratio. Whole blood (340 μL) was re-calcified with 20 μL 0.2M $CaCl_2$ to induce coagulation in a thromboelastogram (TEG) 5000 Analyzer (Hemoscope). n=5-7. Assessed by unpaired, two-tailed Student's t-test. * $P < 0.05$, *** $P < 0.001$. Mean ± SEM. Scale bars: 10mm (**b**)

Supplementary Figure 8

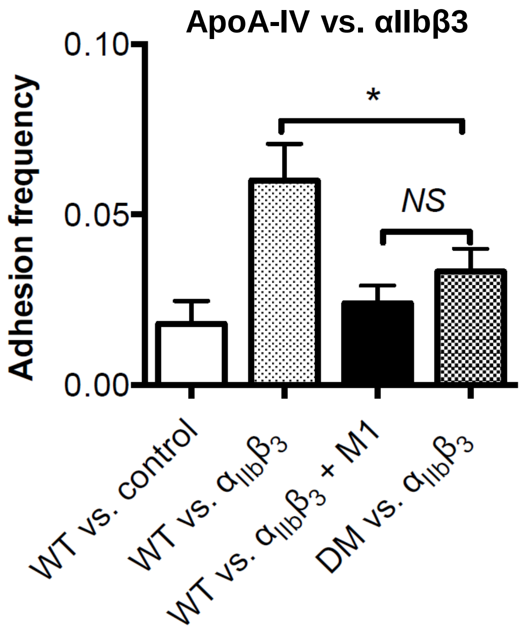

Double mutation at D5 and D13 resulted in the loss of significant binding to αIIbβ3 integrin. Biomembrane-Force-Probe adhesion frequency assays show that double D mutations (DM, i.e. D5 and D13 mutations) abrogated its binding to αIIbβ3 integrin. BSA was used as control. *WT* recombinant apoA-IV, *M1* a specific anti-β3 integrin monoclonal antibody. Assessed by unpaired, two-tailed Student's t-test. NS not significant, * $P < 0.05$. Mean ± SEM. 3 pairs of bead/target each contacted 50 times to estimate an adhesion frequency.

Supplementary Figure 9

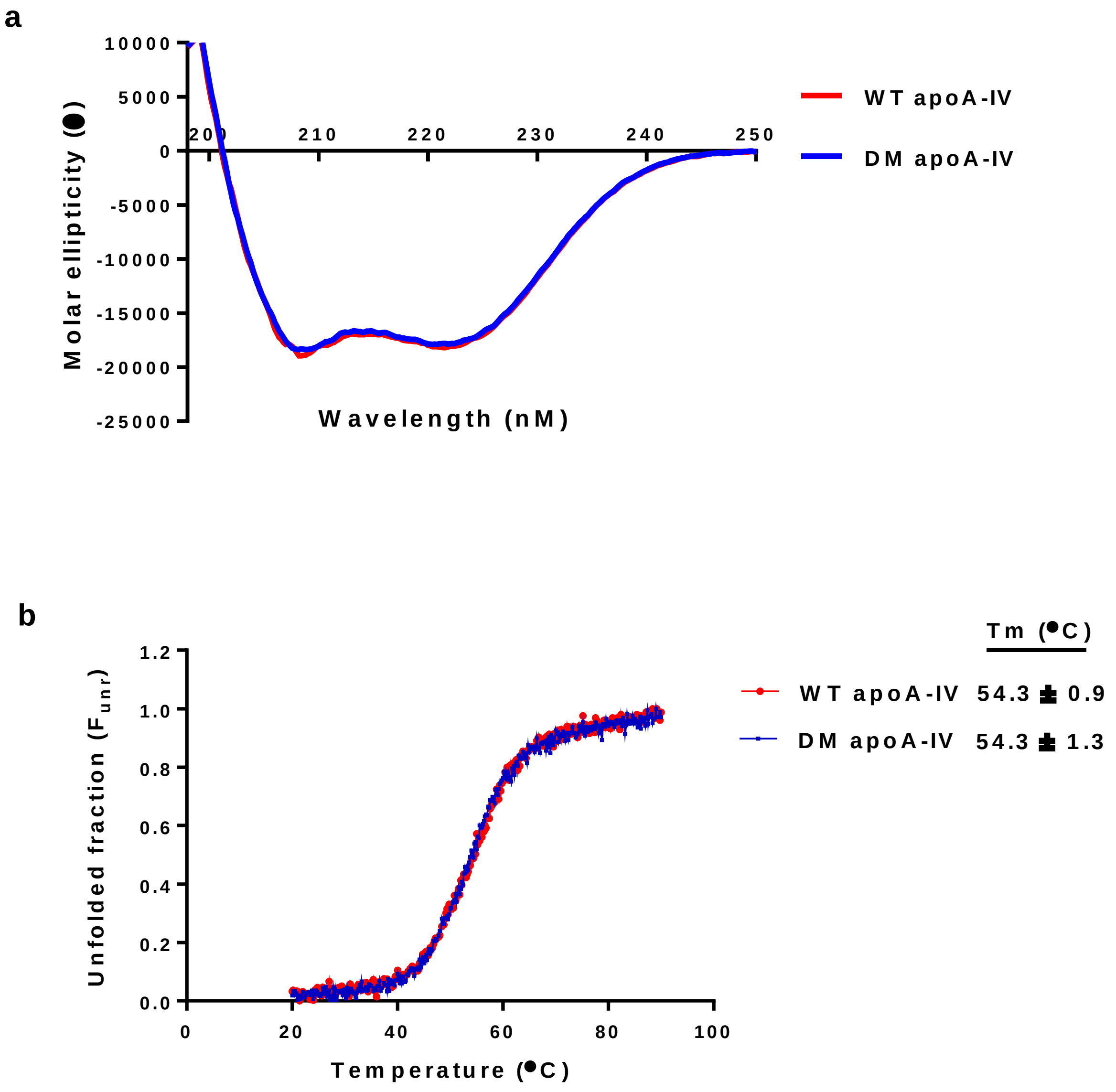

Double aspartic acids mutations at apoA-IV N-terminal does not affect the structure and stability of apoA-IV. a, CD wavelength scans revealed excellent superimposition between the WT and DM apoA-IV CD spectra, suggesting no loss of secondary structural elements as a result of the double D mutations. **b**, Thermal denaturation assays showed that both the WT and DM apoA-IV exhibited similar thermal denaturation profiles with a melting temperature (Tm) of 54.3° C. n=3.

Supplementary Figure 10

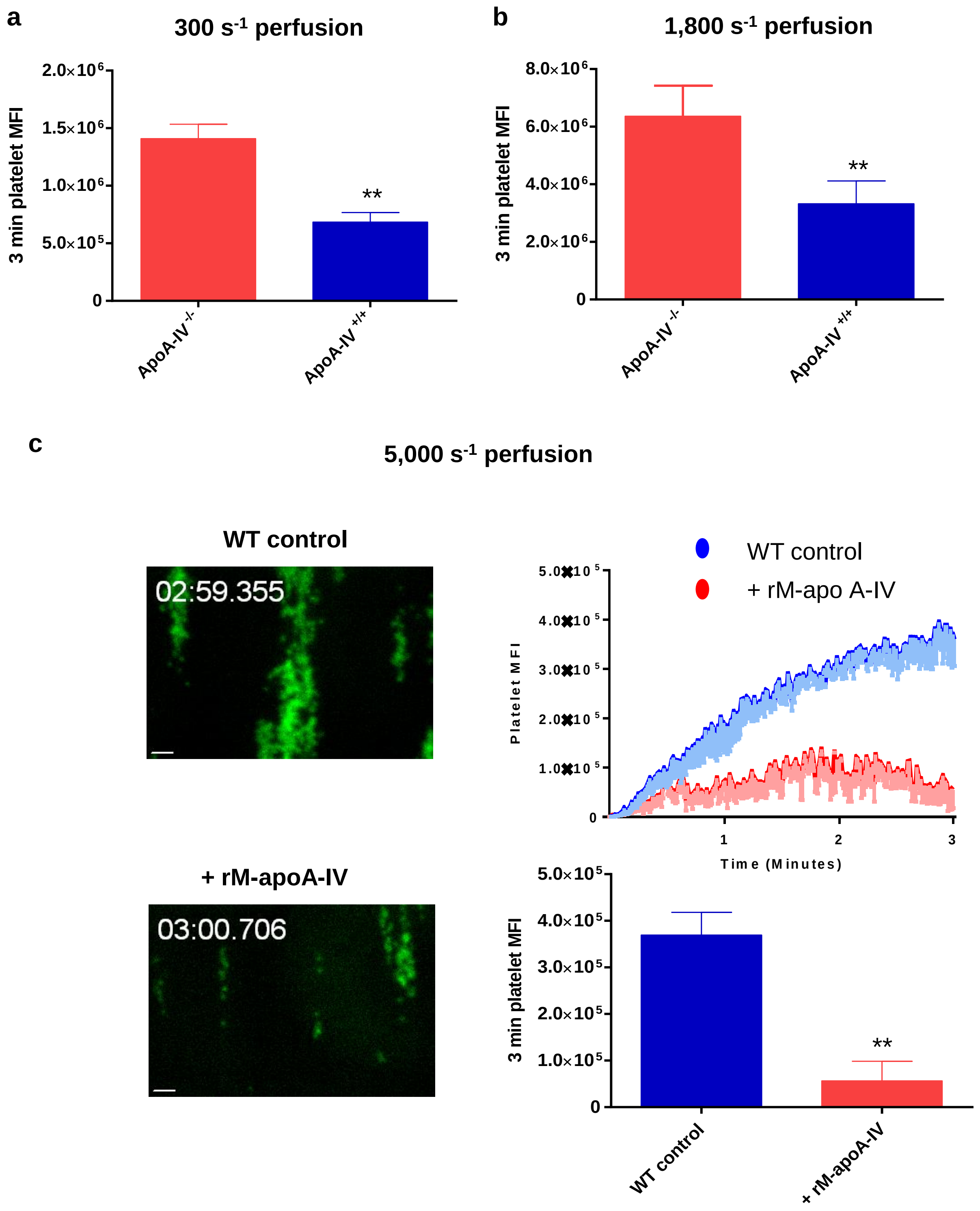

a, b, Enhanced thrombus formation in blood from apoA-IV$^{-/-}$ mice at 3 min under low (**a**) and high (**b**) shear rates. **c,** Inhibition of thrombus formation in blood from WT mice by recombinant mouse apoA-IV (rM-apoA-IV) under stenotic shear rates (n=4 thrombi). Representative images of thrombus formation (green) in four individual experiments were shown. Assessed by unpaired, two-tailed Student's t-test. *** P < 0.01. Mean ± SEM. Scale bars: 10 μm (**c**)

Supplementary Figure 11

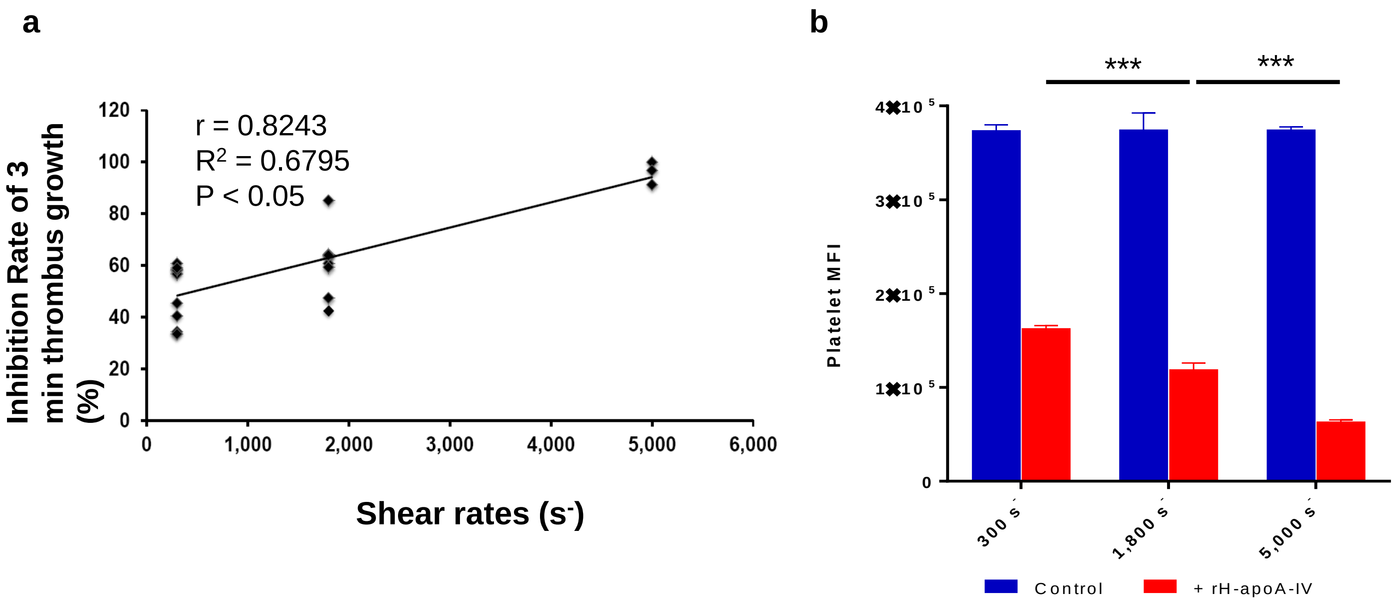

The anti-thrombotic effects of apoA-IV were enhanced with increasing shear rates under flow conditions. Enhanced inhibitory effect of apoA-IV following the increasing shear rates. Inhibitory rates at 3 min perfusion (**a**) and the same volumes of thrombi (i.e. the same platelet MFI in control, **b**) were analyzed. Pearson correlation coefficient r and R^2 were indicated. NS not significant; *** $P < 0.001$. Mean ± SEM.

Supplementary Figure 12

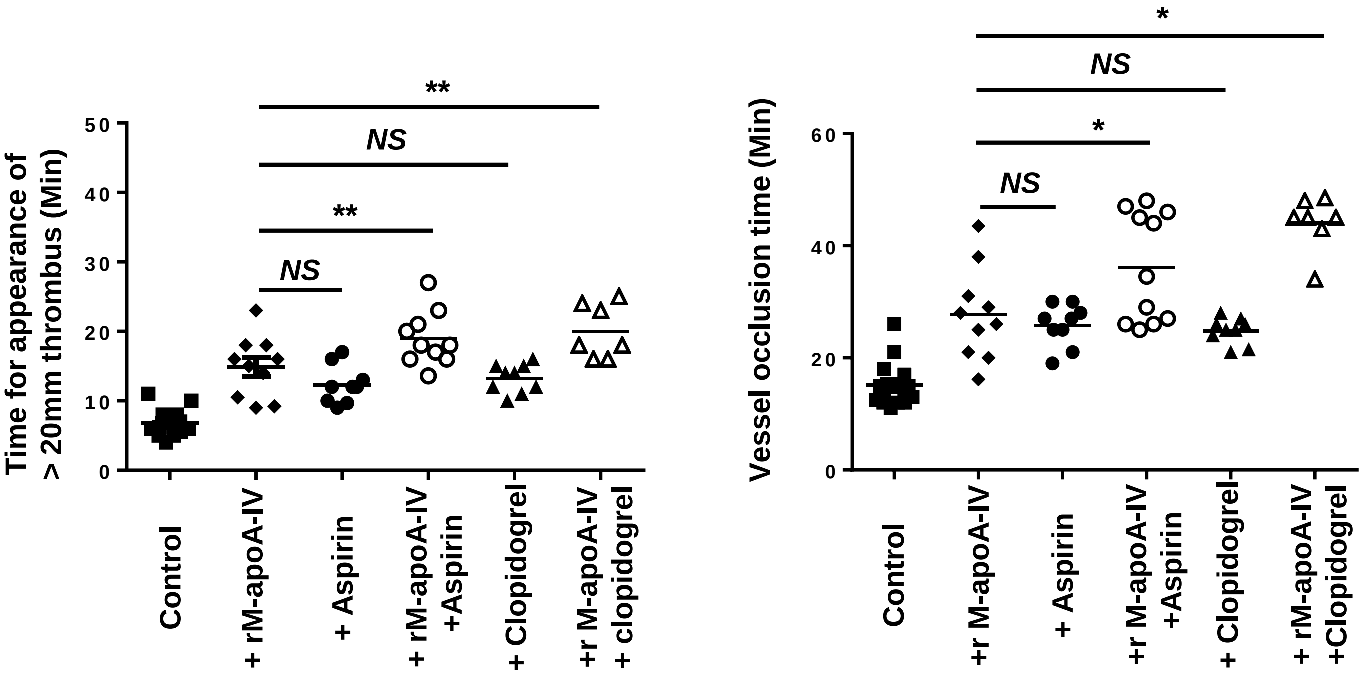

ApoA-IV inhibition of thrombus formation *in vivo* was comparable to aspirin or clopidogrel treatments. Recombinant mouse apoA-IV (rM-apoA-IV, 9 µg/g), aspirin (20 µg/g), and clopidogrel (12 µg/g) were injected into WT mice. Injury was induced by topical application of 30 µL $FeCl_3$ (4%). Thrombus formation including (1) time required for formation of the first 20 µm thrombus, and (2) time to complete vessel occlusion in mesenteric arterioles were evaluated. The inhibitory effect of apoA-IV was similar to aspirin or clopidogrel treatments. Synergism was observed when apoA-IV and aspirin, or apoA-IV and clopidogrel were used in conjunction. n=7-10. NS not significant, * $P < 0.05$, ** $P < 0.01$. Mean ± SEM.

Supplementary Figure 13

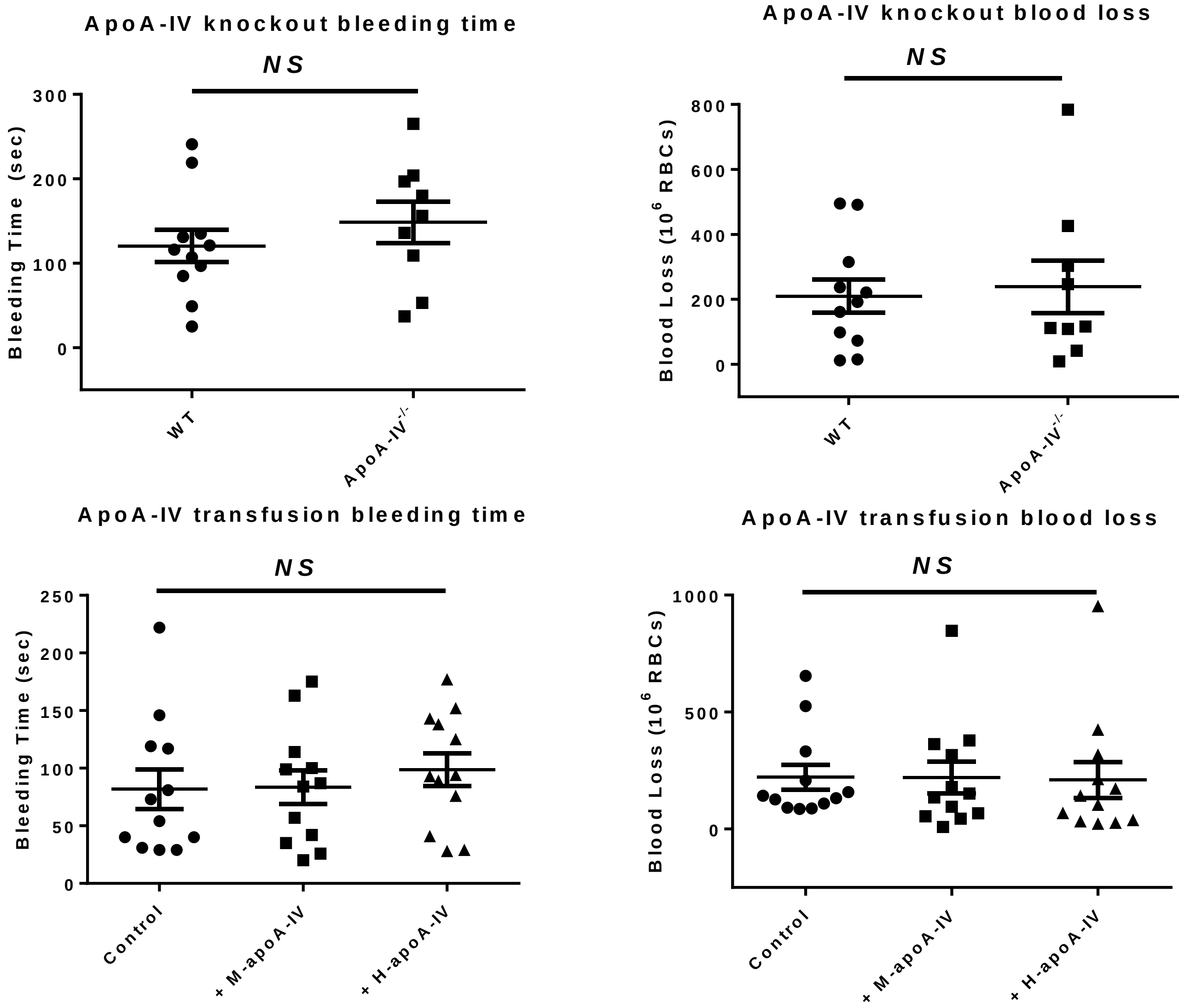

ApoA-IV deficiency or apoA-IV infusion did not alter bleeding time or blood loss. ApoA-IV$^{-/-}$, apoA-IV$^{+/+}$, and WT mice were injected with recombinant apoA-IV where indicated *via* the tail vein 40 min before injury. Bleeding time was recorded as the time to cessation of blood flow (bleeding stopped for >10 s). Blood loss was calculated by counting the red blood cells in the PBS fraction. n=9-12. NS not significant. Mean $\pm$ SEM.

Supplementary Figure 14

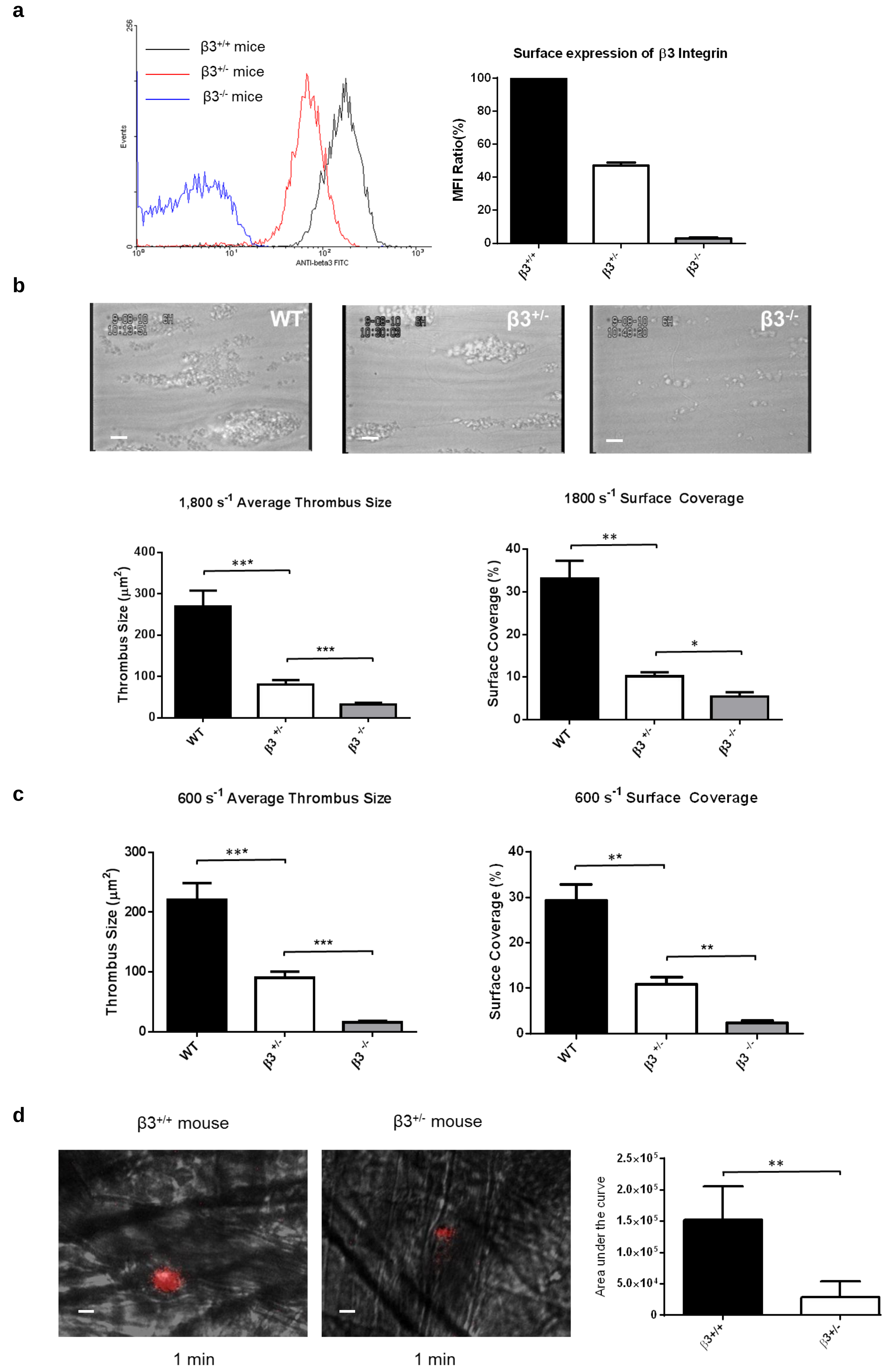

a, **Heterozygous β3 integrin deficiency (β3$^{+/-}$) resulted in markedly decreased thrombus formation *ex vivo* and *in vivo.*** β3 heterozygous (β3$^{+/-}$) mice expressed 50% β3 integrins of WT platelet by flow cytometry (MFI, mean fluorescence intensity). **b,** *Ex vivo* perfusion chamber assay. Blood from WT (β3$^{+/+}$), β3 heterozygous (β3$^{+/-}$), and β3 deficient (β3$^{-/-}$) mice was perfused over type-I collagen-coated chambers under shear rates 1,800 s^{-1} **(b)** and 600 s^{-1} **(c)**. Both average thrombus size and surface coverage were decreased by ~54% in β3$^{+/-}$ whole blood compared with WT. **d**, Thrombus formation (red) in β3 heterozygous (β3$^{+/-}$) mice was significantly decreased in a laser-induced cremaster arteriole thrombosis model. Representative images of thrombus formation in all samples were shown. n=5-7. Assessed by unpaired, two-tailed Student's t-test. * $P < 0.05$; ** $P < 0.01$; *** $P < 0.001$. Mean ± SEM. Scale bars: 10 μm (**b** and **d**)

Supplementary Figure 15

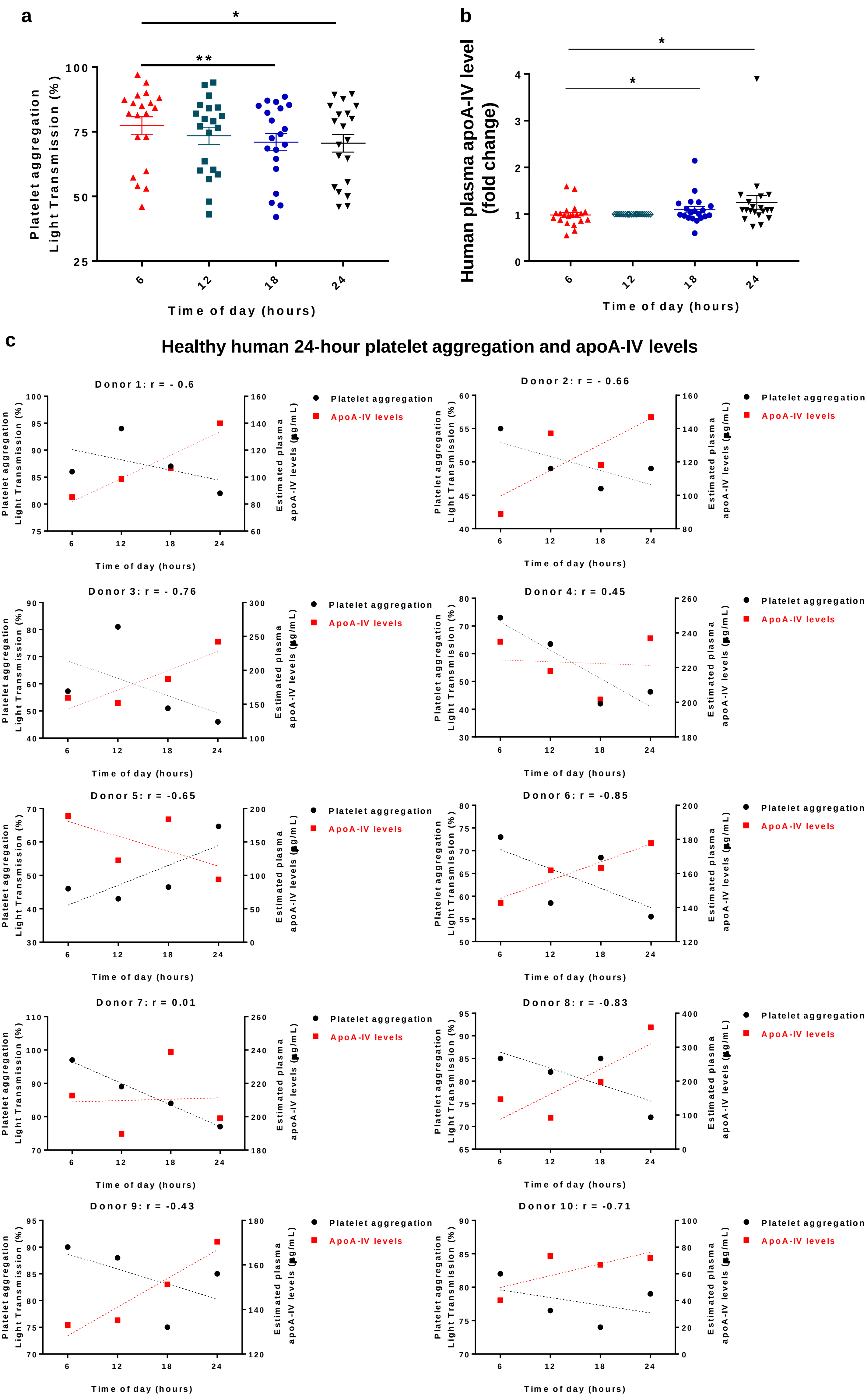

Levels of apoA-IV and platelet aggregation throughout the day are inversely correlated in humans. a, Humans have the strongest platelet aggregation in the morning at 06:00. ADP (6 μM) induced-human platelet aggregation in PRP is shown. **b**, Plasma apoA-IV levels in humans exhibited a midnight peak and nadir in the morning at 06:00. Data are normalized according to apoA-IV level at 12:00. n=20 donors. * $P < 0.05$; ** $P < 0.01$. Mean ± SEM.

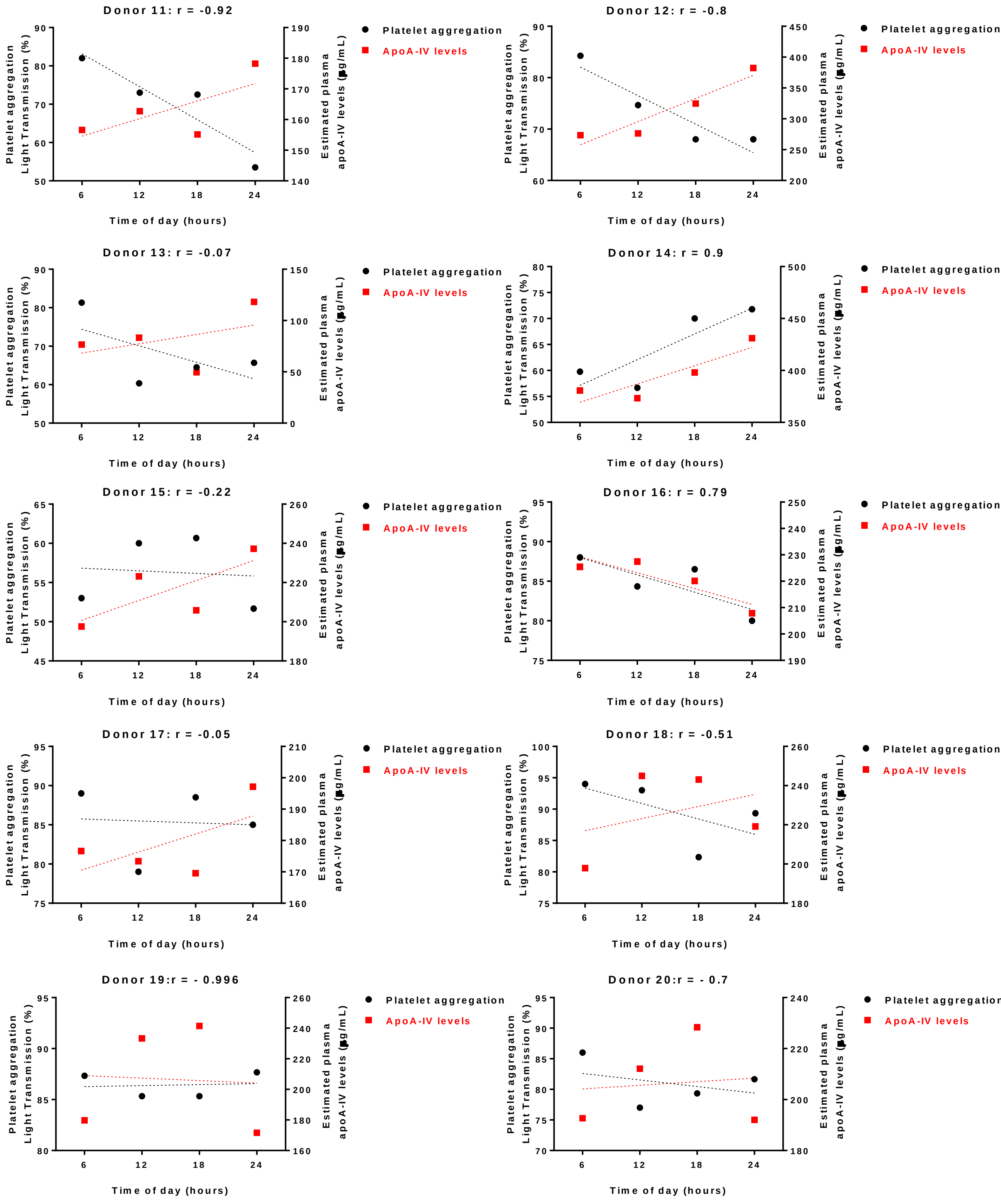

d **Correlation between apoA-IV and platelet aggregation levels throughout the day in 20 donors**

r / numbers	-1 < r < - 0.5	- 0.5 < r < 0	0 < r < 1	Total
	12	4	4	20

c-d, Levels of plasma apoA-IV and platelet aggregation throughout the day in most cases are inversely correlated (12/20 donors: -1 < r <-0.5; 4/20 donors: -0.5 < r < 0; 4/20 donors: 0 < r < 1). The levels of apoA-IV and platelet aggregation in each individual over 24 hours are shown in **c**, with the r value indicates the correlation between apoA-IV and platelet aggregation. r: Pearson correlation coefficient.

Supplementary Figure 16

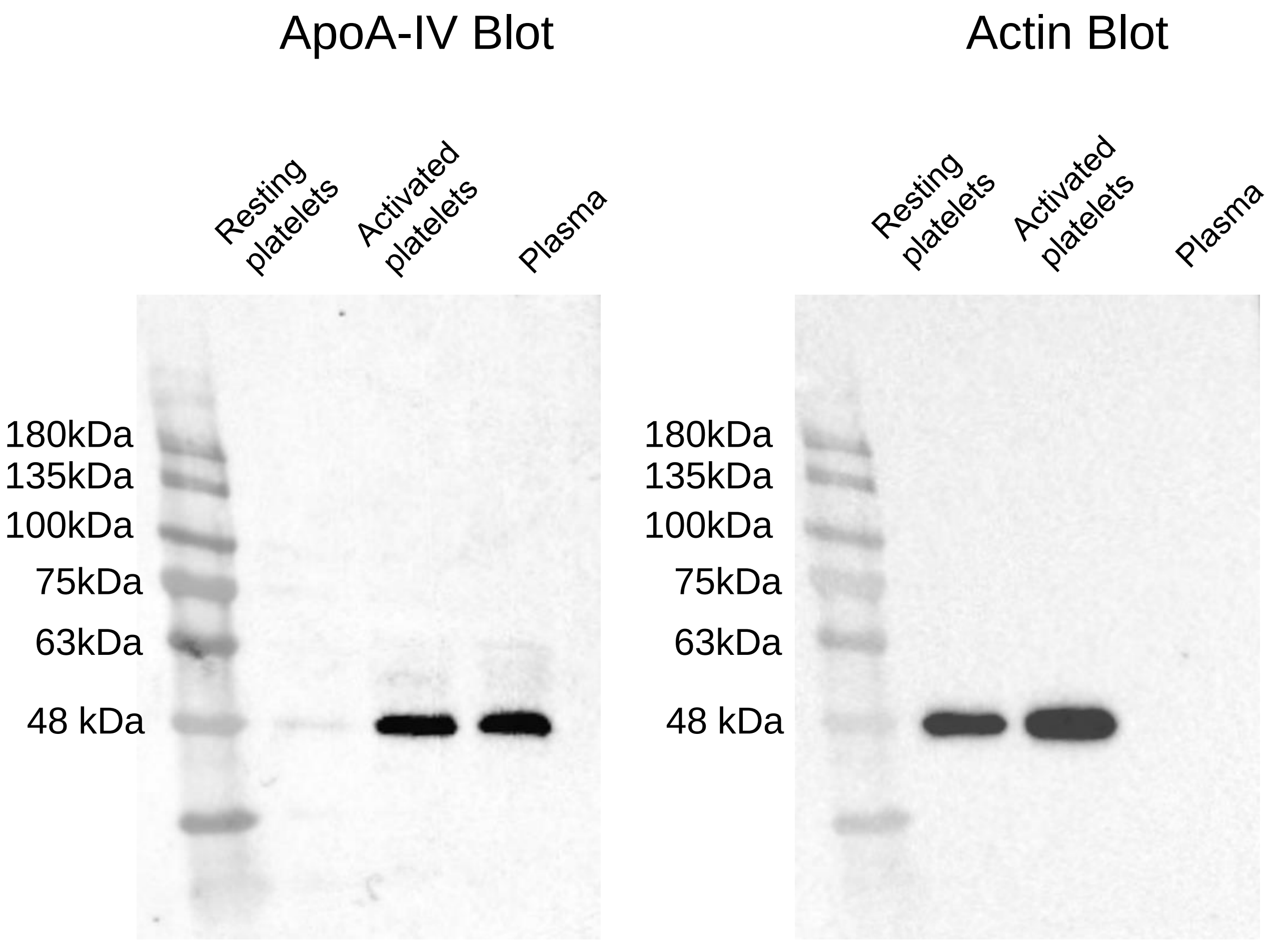

Western blot analysis of platelet bound apoA-IV. Full uncropped western blots from Figure 1b detecting apoA-IV (***left***) and actin (***right***). NP-40 lysed resting platelets, collagen activated platelets and plasma from WT mice were loaded onto a 10% SDS-PAGE gel under reducing conditions. Following transfer onto a nitrocellulose membrane, apoA-IV was detected using goat anti-human apoA-IV antibody (N-20) and then visualized using rabbit anti-goat IgG HRP conjugated antibody. Following apoA-IV detection actin was probed as a loading control. The blots were then stripped using restore stripping buffer and the actin bands were visualized using HRP conjugated goat anti-mouse actin antibody. n=3.

Supplementary Figure 17

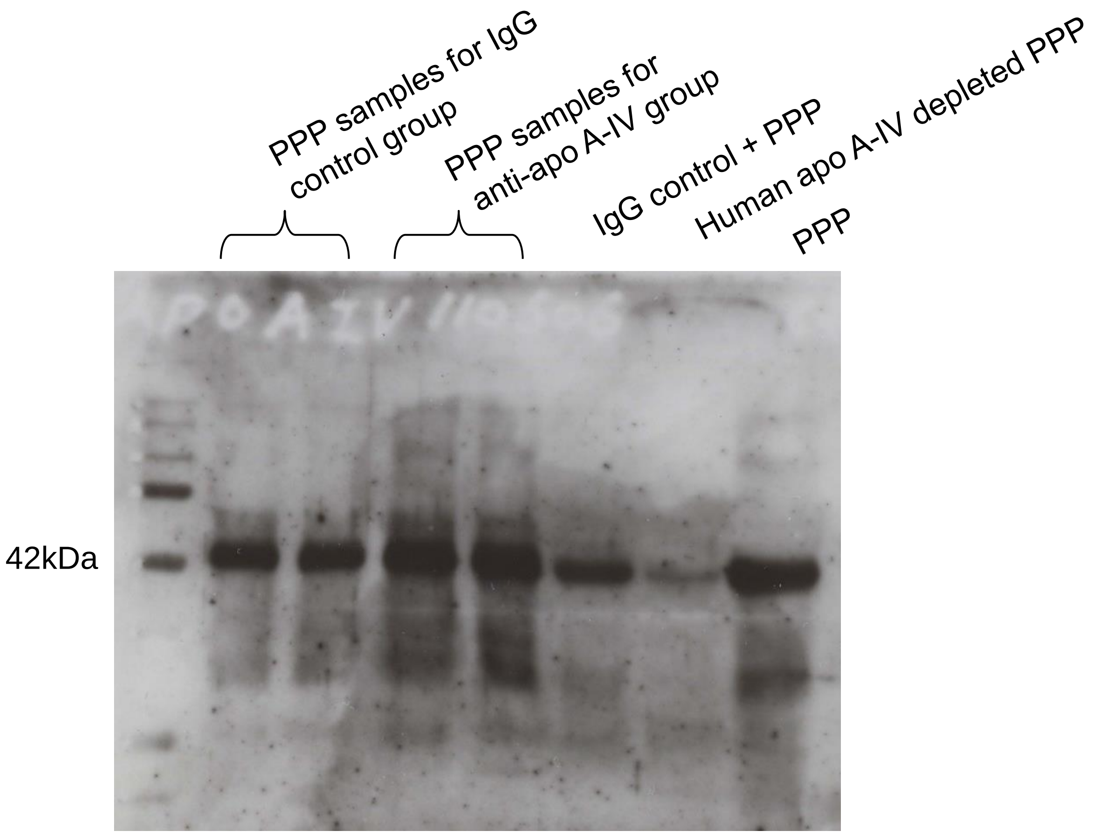

Western blot analysis of human plasma apoA-IV depletion. Full uncropped western blots from Figure 3f detecting human plasma apoA-IV depletion. Goat anti-human apoA-IV antibody (C-20) was coated on the protein G Sepharose beads and the same amount of non-specific goat IgG was used as control. Before immunoprecipitation (IP), human PPP for both groups were prepared (Lane 2-3: PPP samples for IgG control group; Lane 4-5: PPP samples for anti-apoA-IV group) and then added to the beads. The eluate for IgG control group (Lane 6) and apoA-IV depleted group (Lane 7) was collected and used for western blot. ApoA-IV (46kDa) bands were detected. n=3.

Supplementary Table 1

Analyte	Control	+ ApoA-IV
Total cholesterol (mg/dL)	70.05±8.85	67.03±4.89
Triglycerides (mg/dL)	48.31±13.88	40.26±9.17
Phospholipid (mg/dL)	146.36±13.46	139.79±9.63
NEFA (mEq/L)	1.71±0.59	1.97±0.31
HDL cholesterol (mg/dL)	71.43±3.97	70.99±7.61
Non-HDL cholesterol (mg/dL)	18.75±3.43	22.01±2.50
LDL cholesterol (mg/dL)	16.90±2.66	19.54±2.86
VLDL cholesterol (mg/dL)	1.86±0.88	2.46±0.75

Plasma lipid and cholesterol profile in apoA-IV-infused mice were not significantly altered. WT mice injected with either recombinant mouse apoA-IV or buffer alone were anaesthetized and bled *via* the retroorbital plexus into EDTA. Plasma was frozen and sent for analysis of TC, TG, PL, or NEFA, or fresh cold plasma (not frozen) was sent for FPLC analysis of HDL-C, Non-HDL-C, LDL-C, and VLDL-C at the Mouse Metabolic Phenotyping Centre, University of Cincinnati. There were no significant differences in any of these parameters between WT mice and WT mice injected with apoA-IV. n=3.
